# Pharmacokinetics of Mephedrone Enantiomers in Whole Blood after a Controlled Intranasal Administration to Healthy Human Volunteers

**DOI:** 10.3390/ph14010005

**Published:** 2020-12-23

**Authors:** Joanna Czerwinska, Mark C. Parkin, Agostino Cilibrizzi, Claire George, Andrew T. Kicman, Paul I. Dargan, Vincenzo Abbate

**Affiliations:** 1 King’s Forensics, Department of Analytical, Environmental and Forensic Sciences, King’s College London, London SE1 9NH, UK; joanna.czerwinska@kcl.ac.uk (J.C.); MarkParkin@eurofins.co.uk (M.C.P.); andrew.kicman@kcl.ac.uk (A.T.K.); 2 Toxicology Department, Eurofins Forensic Services, Teddington TW11 0LY, UK; 3 Institute of Pharmaceutical Science, King’s College London, London SE1 9NH, UK; agostino.cilibrizzi@kcl.ac.uk; 4 Alere Toxicology (Now Part of Abbott), Abingdon OX14 1DY, UK; claire.george@abbott.com; 5 Clinical Toxicology, Faculty of Life Sciences and Medicine, King’s College London, London SE1 9NH, UK; Paul.Dargan@gstt.nhs.uk; 6 Clinical Toxicology, Guy’s and St Thomas’ NHS Foundation Trust and King’s Health Partners, London SE1 7EH, UK

**Keywords:** mephedrone, enantiomers, whole blood, pharmacokinetics, intranasal administration

## Abstract

Mephedrone, which is one of the most popular synthetic cathinones, has one chiral centre and thus exists as two enantiomers: *R*-(+)-mephedrone and *S*-(−)-mephedrone. There are some preliminary data suggesting that the enantiomers of mephedrone may display enantioselective pharmacokinetics and exhibit different neurological effects. In this study, enantiomers of mephedrone were resolved via chromatographic chiral recognition and the absolute configuration was unambiguously determined by a combination of elution order and chiroptical analysis (i.e., circular dichroism). A chiral liquid chromatography tandem mass spectrometry method was fully validated and was applied to the analysis of whole blood samples collected from a controlled intranasal administration of racemic mephedrone hydrochloride to healthy male volunteers. Both enantiomers showed similar kinetics, however, *R*-(+)-mephedrone had a greater mean C_max_ of 48.5 ± 11.9 ng/mL and a longer mean half-life of 1.92 ± 0.27 h compared with 44.6 ± 11.8 ng/mL and 1.63 ± 0.23 h for *S*-(−)-mephedrone, respectively. Moreover, *R*-(+)-mephedrone had a lower mean clearance and roughly 1.3 times greater mean area under the curve than *S*-(−)-mephedrone. Significant changes in the enantiomeric ratio over time were observed, which suggest that the analytes exhibit enantioselective pharmacokinetics. Even though the clinical significance of this finding is not yet fully understood, the study confirms that the chiral nature, and consequently the enantiomeric purity of mephedrone, can be a crucial consideration when interpreting toxicological results.

## 1. Introduction

**Figure 1:**
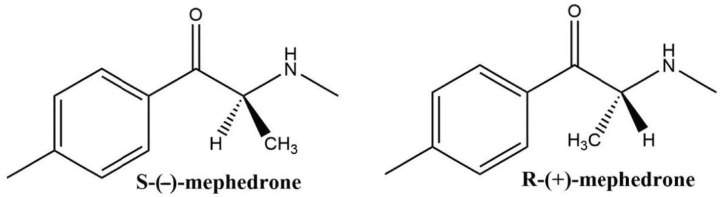
Chemical structure of mephedrone enantiomers.

Mephedrone (4-methylmethcathinone) is a popular synthetic cathinone which is structurally and chemically similar to other stimulant drugs, such as amphetamine [1]. With a single chiral centre at the α-carbon, mephedrone exists as two enantiomers: *R*-(+)-mephedrone (R-MEPH) and *S*-(−)-mephedrone (S-MEPH) [2], as shown in Figure 1.

Even though recreational use of mephedrone in Europe began in 2009, its synthesis was first described in 1929 [3]. The main synthetic route involves α-bromination of 4-methylpropiophenone and a subsequent reaction with methylamine hydrochloride and triethylamine. The resulting mixture is treated with gaseous or aqueous hydrochloric acid and is recrystallised to yield mephedrone hydrochloride [4]. This method produces a racemic mixture but a stereoselective synthesis via Friedel–Crafts acylation can also be performed [5]. Even though stereoselective synthesis is possible, it is thought that “street mephedrone” has been primarily sold as a racemic mixture [6], which is most likely attributed to the simplicity of the synthesis and the availability of the precursors.

Enantiomers usually differ in their biochemical activity which may result in one enantiomer being biologically active while the other may be inactive or may even produce adverse effects [7]. To our knowledge, there are no published reports available on pharmacokinetics of mephedrone enantiomers in humans, but preliminary data derived from both in vitro experiments, animal studies and analysis of human urine suggest that mephedrone enantiomers may have a different metabolic pathway and exhibit different neurological effects [2,8,9,10]. Castrignanò et al. found that R-MEPH was the predominant analyte in in vitro experiments performed in human liver microsomes and in pooled human urine collected from music festivals [2]. By contrast, a different study that investigated the presence of 56 biomarkers (including mephedrone) in wastewater found samples to be enriched with S-MEPH [9]. A study in rats has shown that R-MEPH results in more stimulant-like effects than S-MEPH due to its higher selectivity for dopaminergic system [8]. Moreover, the phase 1 metabolites of mephedrone possess bioactive properties, with the stereoisomers inhibiting dopamine, norepinephrine and serotonin transport-mediated uptake. Of note is that the S-enantiomers of nor-mephedrone and 4-hydroxytolyl-mephedrone have been reported to be several times more potent as inhibitors of serotonin mediated uptake than the corresponding R-enantiomers [10]. All in all, assessing the degree of asymmetry in the abundance of the enantiomers of mephedrone and its metabolites in biological samples should hopefully add insight into the pharmacodynamics of the drug.

In a recently published study describing detection of racemic mephedrone in human whole blood and plasma, mephedrone was detected for 6 h post administration and showed rapid absorption and elimination [11]. Herein, we report the first in-depth investigation of pharmacokinetic profiles of mephedrone enantiomers derived from whole blood analysis following a controlled intranasal administration of 100 mg of pure racemic mephedrone hydrochloride to healthy human volunteers.

## 2. Results

### 2.1. Method Validation

The results of method validation are presented in Supplementary Materials. Briefly, intra- and inter-day precision were within ±15% of the true value (Table S2 in Supplementary Materials). Mean linearity of *R*^2^ > 0.999 was achieved for both enantiomers in all three validation runs (Figure S1 and Table S1 in Supplementary Materials). Matrix effect ranged from 97.5 ± 5.2% to 102 ± 7.7% for both enantiomers (Table S3 in Supplementary Materials). Carryover was not observed.

### 2.2. Determination of the Elution Order

**Figure 2:**
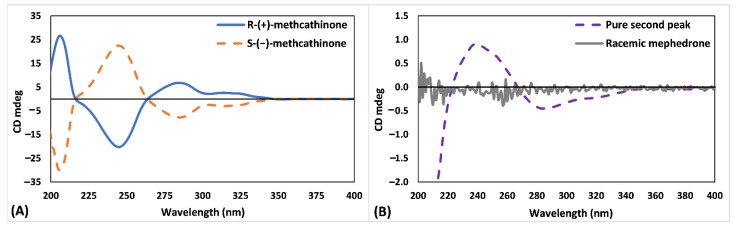
Circular dichroism profiles of (**A**) pure S-(−)-methcathinone and R-(+)-methcathinone and (**B**) racemic mephedrone and the second eluting peak from (±)-mephedrone samples (i.e., S-(−)-mephedrone).

Alongside collected fractions, 1 mg/mL solutions of pure methcathinone enantiomers were analysed by circular dichroism (CD) spectroscopy. Figure 2 shows the CD spectrum of *S*-(−)-methcathinone and *R*-(+)-methcathinone (Figure 2A, mirror-image CD profiles) and the second eluting peak of (±)-mephedrone samples (see Figures S3 and S4 in Supplementary Materials for the chiral chromatographic separation). The CD spectrum of the second eluting peak produces a broad positive Cotton effect between 220 and 270 nm (Figure 2B), fully matching the positive peak recorded for *S*-(−)-methcathinone. Therefore, (S)-absolute configuration can be confidently assigned to the second eluting peak collected from (±)-mephedrone samples. Indirectly, this result also confirms that the first eluting peak of (±)-mephedrone is the *R*-(+)-mephedrone, a conclusion based on the structural homology between mephedrone and methcathinone.

### 2.3. Concentrations of Mephedrone Enantiomers in Whole Blood

**Figure 3:**
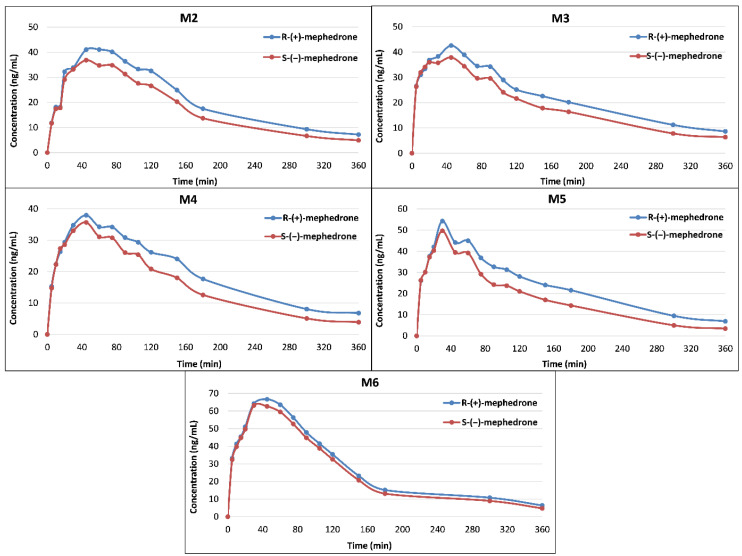
Concentrations of R-(+)-mephedrone and S-(−)-mephedrone in whole blood in five participants (M2–M6).

In order to highlight individual changes in analyte concentration, Figure 3 shows concentration profiles of S-MEPH and R-MEPH in each participant (M2–M6) up to 6 h post administration. R-MEPH and S-MEPH were detected in whole blood between 5 and 360 min, but R-MEPH reached higher concentrations between 20 and 360 min in all participants. Analytes were not detectable on Day 2 or Day 3.

### 2.4. Enantiomeric Ratio (ER)

ER, used to show the proportion of enantiomers in a mixture [12], was determined by the relative percentages of the peak area ratios according to Equation (1) [13].

Equation (1). Equation for calculating enantiomeric ratio.

(1) $$\mathrm{Enantiomeric}\;\mathrm{ratio}=\frac{peak\;area\;ratio\;of\;R-MEPH}{peak\;area\;ratio\;of\;S-MEPH}$$

**Figure 4:**
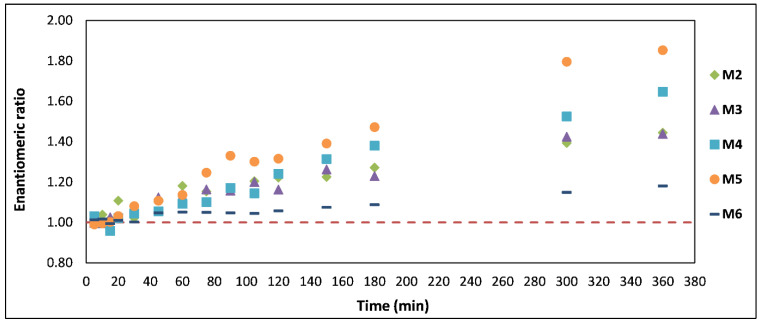
Changes in the enantiomeric ratio over time post mephedrone administration in M2–M6; the dashed line at 1.0 shows the expected enantiomeric ratio if both enantiomers were present in whole blood samples at 1:1 ratio.

ER is expected to be 1.0 if both enantiomers are present in a matrix at 1:1 ratio (i.e., racemic mixture). ER calculated for each participant at each timepoint between 5 and 360 min is presented in Figure 4. According to the one sample *t*-test, statistically significant changes from the expected value of 1.0 start to emerge at 45 min (mean ER ± SD = 1.09 ± 0.04, *p*-value: 0.005) and continue until 360 min (mean ER ± SD = 1.51 ± 0.25, *p*-value: 0.011). Calibration standards and quality control samples had mean ER ± SD of 0.992 ± 0.056 across all runs.

### 2.5. Pharmacokinetic Analysis

Whole blood drug concentrations were fitted with a single-dose first-order elimination phase model and calculated pharmacokinetic parameters are summarised in Table 1.

Both enantiomers showed similar kinetics but S-MEPH peaked earlier at 39 ± 8 min and had shorter t_1/2_ of 1.63 ± 0.23 h compared to 45 ± 11 min and 1.92 ± 0.27 h for R-MEPH, respectively. Differences in the area under the curve (AUC) and clearance (CL) were also observed. R-MEPH resulted in roughly 1.3 times greater mean AUC compared to S-MEPH and had lower mean clearance of 126 ± 22 mL min^−1^ kg^−1^ compared with 164 ± 29 mL min^−1^ kg^−1^ for the other enantiomer. Mean V_d_ was similar for R-MEPH (21.0 ± 4.5 L kg^−1^) and S-MEPH (23.1 ± 4.6 L kg^−1^).

Paired *t*-test was performed to check if the pharmacokinetic parameters obtained for R-MEPH and S-MEPH were significantly different. At 95% confidence level, *C*_max_, *k*_el_, *t*_1/2_, AUC, CL and *V*_d_ were all statistically different, with the two-tailed *p*-value of 0.001, 0.004, 0.011, 0.002, 0.005, 0.002, respectively. The only parameter which did not show statistically significant difference was T_max_ (*p*-value of 0.178).

## 3. Discussion

The whole blood concentrations of R-MEPH reached a higher C_max_, had a greater AUC and a longer t_1/2_ than S-MEPH in each of the participants. These results are in agreement with previous research that found R-MEPH to be a predominant analyte in pooled human urine samples and in in vitro experiments performed in human liver microsomes [9]. In the pharmacodynamic context, a study in rats has shown that R-MEPH rather than S-MEPH results in more stimulant-like effects due to its 50-fold greater selectivity for dopaminergic system [8]. R-MEPH was also found to be responsible for increased intracranial self-stimulation, demonstrating greater abuse-related effects [8]. “Street mephedrone” is thought to be sold as a racemic mixture, which has been primarily attributed to the affordability and availability of the precursors [6]. Should the starting material become unavailable, expensive or subject to strict international control, an alternative synthetic route (perhaps enantiomerically specific) could be employed. If the resulting synthetic product contains enantiomeric excess of R-MEPH, mephedrone users may be at risk of greater toxicity.

Recently published [11] mean racemic mephedrone concentrations in whole blood were approximately two times higher than the mean concentrations of each individual enantiomer. R-MEPH had more comparable pharmacokinetic parameters to those obtained for racemic mephedrone in whole blood. Both peaked at approximately 50 min, had the same *k*_el_ of 0.006 ± 0.001 min^−1^ and nearly identical *t*_1/2_ of 2.12 ± 0.33 h for the racemic mephedrone and 1.92 ± 0.27 h for R-MEPH. *C*_max_ of 101 ± 45.4 ng/mL was observed for racemic mephedrone, which was 2.26 times greater than the *C*_max_ of S-MEPH and 2.08 times greater than the *C*_max_ of R-MEPH.

Changes in the ER over time showed whole blood samples collected between 45 and 360 min to have a statistically different ER from the value of 1.0. Differences in concentrations of S-MEPH and R-MEPH may be a result of pharmacokinetic processes occurring at different rates during drug absorption, distribution, metabolism or excretion [14]. Some drugs can be chemically or biochemically inverted in vivo in a unidirectional or bidirectional manner [15]. Moreover, polymorphic drug metabolism, gender, age, disease state and medications could all affect concentrations of enantiomers in whole blood, although given the design of this study, polymorphic drug metabolism is likely to play the most significant role. Hepatic cytochrome P450 2D6 (CYP2D6), responsible for mephedrone metabolism, is subject to genetic polymorphism which causes variations in CYP2D6 enzymatic activity [16]. Participants in our study were not genotyped for CYP2D6 polymorphism but it has been demonstrated that significantly altered mephedrone plasma concentrations can be a result of CYP2D6 activity, with those who have no or low CYP2D6 functionality being at greater risk of acute toxicity [17].

Even though chiral analysis is considered important and sometimes mandatory for accurate interpretation of analytical findings [18], it is yet to be widely adopted as part of routine procedures in clinical and forensic toxicology. In this context, it may be worth following-up routine analysis of blood samples where mephedrone has been detected with chiral analysis. A small number of case studies could be initially performed to understand the usefulness of this additional information, in particular with reference to R-MEPH.

## 4. Materials and Methods

### 4.1. Reagents

Racemic mephedrone hydrochloride, racemic mephedrone-d_3_ hydrochloride (MEPH-d_3_), *S*-(−)-methcathinone and *R*-(+)-methcathinone were purchased as certified reference materials from Sigma-Aldrich (Dorset, UK). Racemic mephedrone hydrochloride in powder form used for the human administration was purchased from Chiron (Trondheim, Norway). The chemical structure, purity and racemic nature of the powder was confirmed by mass spectrometry, circular dichroism (CD) spectroscopy and nuclear magnetic resonance.

All solvents were HPLC grade unless stated otherwise. Methanol (MeOH; HPLC grade and Optima LC/MS grade), acetonitrile (ACN), isopropyl alcohol (IPA), dichloromethane (DCM), sodium phosphate monobase, sodium phosphate diabase, formic acid, acetic acid and ammonium hydroxide (0.88 S.G., 35%) were purchased from Fisher Scientific (Loughborough, UK). Diethylamine (DEA) and ethanol (EtOH; LiChrosolv grade) were purchased from Sigma-Aldrich (Dorset, UK). Ultrapure water (18 MΩcm) was prepared on an ELGA Purelab Maxima HPLC water purification system (High Wycombe, UK). Xtrackt^®^ DAU High Flow (150 mg, 3 mL) cartridges were purchased from Chromatography Direct (Runcorn, UK).

### 4.2. Blank Matrix

Drug-free whole blood was collected by trained phlebotomists into 5 mL Vacutainers^®^ (BD, Worthing, UK) containing 12.5 mg of sodium fluoride and 10 mg of potassium oxalate (NaF/KOx).

Ethical approval for the collection of drug-free matrix was granted by the Research Ethics Committee at King’s College London (HR 16/17 4237).

### 4.3. Volunteer Administration Study and Sample Collection

All subjects gave their informed consent for inclusion before they participated in the study. The study was conducted in accordance with the Declaration of Helsinki, and the protocol was approved by the Riverside National Research Ethics Service (16/LO/1342). Details of the administration study have been recently published [11]. Briefly, participants enrolled into the study were occasional users of mephedrone or other stimulant drugs, but were drug-free 1 week before the mephedrone administration, which was verified by urine analysis (performed using a validated ultra-high-performance liquid chromatography–high-resolution mass spectrometry method). Six healthy male volunteers nasally insufflated 100 mg of mephedrone hydrochloride supplied as a racemic mixture (purity: 96.3 ± 0.5%). Whole blood (5 mL) was then collected into Vacutainers^®^ containing NaF/KOx preservative at 0 min (before administration on Day 1), 5 min, 10 min, 15 min, 20 min, 30 min, 45 min, 60 min, 75 min, 90 min, 105 min, 2 h, 2.5 h, 3 h, 5 h, 6 h, Day 2 and Day 3. Samples were stored at +4 °C until analysis.

Whole blood samples were collected from six participants but for the purpose of this study, samples from five participants (referred to here as M2–M6) were analysed. Whole blood samples from participant M1 were discarded due to stability concerns [19] as they had been collected five months before the chiral analysis commenced.

### 4.4. Working Solutions

Working solutions, used for the preparation of the calibration curve, contained mephedrone only and were made in MeOH:water (50:50 *v*/*v*) at 160, 200, 400, 700, 1000, 2000, 4000 ng/mL. Working solution used for the preparation of the quality control (QC) samples at low (Low), medium (Med) and high (High) levels were also made in the same solvent at 200, 800, 3000 ng/mL, respectively. Internal standard (IS) solution, used for quantification of analytes in the samples, contained racemic MEPH-d_3_ at 50 ng/mL in MeOH:water (50:50 *v*/*v*).

### 4.5. Calibration Standards and Quality Control Samples

Matrix-matched calibration standards containing racemic mephedrone at 8, 10, 20, 35, 50, 100, 200 ng/mL were prepared by the addition of an appropriate volume of the working solution to whole blood. QC Low at 10 ng/mL, QC Med at 40 ng/mL and QC High at 150 ng/mL were prepared in the same way. As these racemic calibration standards and QCs were separated into two enantiomers (i.e., two chromatographic peaks), half of their concentration was used for plotting calibration curves and assessing the accuracy of the QCs. Calibration curves were constructed by plotting concentration against peak area ratio (i.e., peak area of each enantiomer divided by the peak area of the corresponding enantiomer of the IS).

Calibration standards and QCs were prepared fresh on the day of sample analysis.

### 4.6. Sample Preparation

Sample extraction has been described before [19]. Briefly, 100 µL of whole blood was taken through solid phase extraction using mixed-mode cartridges. Samples were eluted with 4 × 1 mL of DCM:IPA:ammonium hydroxide (78:20:2 *v*/*v*/*v*) and dried under nitrogen at 50 °C. Samples were then reconstituted with 100 µL of 0.1% DEA in EtOH:MeOH (20:80 *v*/*v*).

### 4.7. Instrumentation

Sample analysis was performed by liquid chromatography–tandem mass spectrometry using a Xevo TQ-S triple quadrupole mass spectrometer (MS; Waters, Manchester, UK) coupled to an Acquity ultra performance liquid chromatograph system (Waters, Manchester, UK). Optimised MS conditions have been published elsewhere [19].

Mephedrone and MEPH-d_3_ were monitored using selected reaction monitoring (SRM) as detailed in Table 2. Chromatographic separation was performed on a 150 mm × 3 mm, 3 µm CHIRALPAK^®^ AD-3 column (Illkirch, France) held at room temperature. The flow rate was 0.1 mL/min with 0.1% DEA in EtOH:MeOH (20:80 *v*/*v*) as mobile phase used in an isocratic mode. The total run time was 10 min.

### 4.8. Determination of the Elution Order

To determine the elution order of the enantiomers, fractions obtained from a semi-prep chiral chromatography injection were collected and analysed by CD. Pure mephedrone enantiomers were not commercially available at the time of this research, therefore, the chemical analogues, *R*-(+)-methcathinone and *S*-(−)-methcathinone, were analysed by CD alongside fractions of mephedrone. Methcathinone differs from mephedrone by the absence of the 4-methyl group on the benzene ring (see Figure S2 in Supplementary Materials for methcathinone structure). Since the remaining structure, including the chemical environment surrounding the chiral centre, is identical, CD spectra of mephedrone fractions were compared with the reference CD spectra of methcathinone enantiomers, which have established an absolute configuration [20,21].

To isolate pure enantiomers in milligram quantities, racemic mephedrone was injected onto a 250 × 10 mm, 5 µm Lux^®^ Amylose-1 (Phenomenex, UK) semi-preparative column (which provides the same selectivity as CHIRALPAK^®^ AD-3 used for sample analysis). The separation was performed on Agilent HPLC 1050 system (Agilent, UK) equipped with a manual injector coupled to a diode array detector. The column was held at room temperature and 0.1% DEA in EtOH:MeOH (20:80 *v*/*v*) was used as mobile phase in an isocratic mode. An injection of a mephedrone solution prepared at 5 mg/mL was performed. To check the purity of collected fractions, fractions were diluted 1 in 10 with mobile phase and injected on the analytical CHIRALPAK^®^ AD-3 column (150 mm × 3 mm, 3 µm). Since one enantiomer is sufficient to establish the configuration of both, only one peak (i.e., the second eluting peak) was isolated and characterised. Pure fractions were then dried overnight under vacuum at room temperature, dissolved in MeOH and combined.

CD spectroscopy (Photophysics Chirascan Plus spectrometer, Leatherhead, UK) was used to analyse the combined fraction. One-millimetre Quartz Suprasil rectangular cells (Hellma, UK) were used in the region of 450–180 nm. The instrument was flushed continuously with pure evaporated nitrogen throughout the experiment. The following parameters were employed: 2 nm spectral bandwidth, 1 nm stepsize and 1.0 s accumulation time per point. The CD spectra were solvent baseline corrected and measured at +23 °C. The CD spectra were smoothed using the Savitzky–Golay method for better presentation. Data processing was done using APL Prodata Viewer (version 4.2.15) and spectra were modified in Origin (version 6.0). The intensities are presented as ellipticity values (mdeg).

### 4.9. Pharmacokinetic Calculations

Pharmacokinetic data were determined by using a non-compartmental pharmacokinetics data analysis software (PK Solutions, Summit Research Services, Colorado (version 2.0)).

Peak concentration (*C*_max_) and the time after dosing when it occurred (*T*_max_) were observed directly from the data. The elimination rate constant (*k*_el_) was calculated using a log-linear regression of the elimination phase, which was achieved by plotting analyte concentration versus time and using data points which produced r^2^ of at least 0.97. Elimination half-life (*t*_1/2_) was calculated as ln(2)/*k*_el_. Clearance (CL) was calculated as dose/AUC. Volume of distribution (*V*_d_) was calculated as CL/*k*_el_. Both CL and *V*_d_ were adjusted for the individual’s weight and are reported here as apparent CL and *V*_d_ because absolute bioavailability of mephedrone is unknown.

### 4.10. Statistical Analysis

One sample t-test and paired *t*-test were calculated using GraphPad Prism (version 7.0). The data set (*n* = 5) at each time point for the concentrations of mephedrone were normally distributed according to the D’Agostino–Pearson normality test (*p*-value > 0.05).

### 4.11. Validation Procedure

Validation experiments determined selectivity, linearity, inter- and intra-day precision and accuracy, limit of detection (LOD), lowest limit of quantification (LLOQ), recovery, matrix effect and carryover. The method was validated for mephedrone according to the Food and Drug Administration guidelines [22] and recommendations published by Peters et al. [23]. Validation experiments are described in Supplementary Materials.

## 5. Conclusions

In this study, enantiomers of (±)-mephedrone were quantified in human whole blood samples from a controlled drug administration. Mephedrone exhibited enantioselective pharmacokinetics whereas R-MEPH reached a higher C_max_, had a greater AUC and longer t_1/2_.

## Figures and Tables

**Table 1:** Pharmacokinetic data from the analysis of R-(+)-mephedrone (R-MEPH) and S-(−)-mephedrone (S-MEPH) in whole blood samples from five male participants (M2–M6).

	**R-MEPH**						
	***C*** **_max_**	***T*** **_max_**	***k*** **_el_**	***t*** **_1/2_**	**AUC**	**CL**	***V*** **_d_**
	**(ng/mL)**	**(min)**	**(min^−1^)**	**(h)**	**(ng mL^−1^ h)**	**(mL min^−1^ kg^−1^)**	**(L kg^−1^)**
**M2**	41.1	60	0.006	1.87	192	114	18.5
**M3**	42.5	45	0.005	2.3	209	122	24.4
**M4**	38	45	0.006	2	179	157	27.2
**M5**	54.2	30	0.006	1.89	201	100	16.5
**M6**	66.7	45	0.007	1.56	219	137	18.6
**Mean ± SD**	48.5 ± 11.9	45 ± 11	0.006 ± 0.001	1.92 ± 0.27	200 ± 16	126 ± 22	21.0 ± 4.5
	**S-MEPH**						
	***C*** **_max_**	***T*** **_max_**	***k*** **_el_**	***t*** **_1/2_**	**AUC**	**CL**	***V*** **_d_**
	**(ng/mL)**	**(min)**	**(min^−1^)**	**(h)**	**(ng mL^−1^ h)**	**(mL min^−1^ kg^−1^)**	**(L kg^−1^)**
**M2**	36.8	45	0.007	1.69	150	146	21.4
**M3**	37.9	45	0.006	2.01	168	153	26.6
**M4**	35.7	45	0.007	1.57	130	216	29.4
**M5**	49.7	30	0.008	1.45	136	148	18.6
**M6**	63.2	30	0.008	1.44	191	158	19.7
**Mean ± SD**	44.6 ± 11.8	39 ± 8	0.007 ± 0.001	1.63 ± 0.23	155 ± 25	164 ± 29	23.1 ± 4.6

**Table 2:** The retention time, selected reaction monitoring (SRM) transitions and collision energy for S-MEPH and R-MEPH.

Analyte	Retention Time (min)	Precursor ion (*m*/*z*)	Product Ion (*m*/*z*)	Collision Energy (V)	Internal Standard
**S-MEPH**	5.9	160.4 **	145.1 *	15	S-MEPH-d_3_
			144.1	33	
			91.1	28	
**R-MEPH**	4.9	160.4 **	145.1 *	15	R-MEPH-d_3_
			144.1	33	
			91.1	28	
**S-MEPH-d_3_**	5.9	163.4 **	148.4	19	
**R-MEPH-d_3_**	4.9	163.4 **	148.4	19	

* Quantifying transition, ** dehydrated precursor ion.

## Data Availability

The data presented in this study are available on request from the corresponding author. The data are not publicly available due to privacy restrictions.

## Supplementary Materials

The following are available online at https://www.mdpi.com/1424-8247/14/1/5/s1, Figure S1. Example calibration curves obtained for R-MEPH and S-MEPH. Table S1. LOD, LLOQ, calibration range and calibration parameters for R-MEPH and S-MEPH in human whole blood (NaF/KOx); Table S2. Precision and accuracy at QC Low, QC Med and QC High for R-MEPH and S-MEPH in human whole blood (NaF/KOx); * average value of 18 measurements over 3 days; Table S3. Analyte recovery and matrix effect for R-MEPH and S-MEPH at QC Low and QC High in human whole blood (NaF/KOx); Figure S2. Chemical structure of (±)-mephedrone and (±)-methcathinone; Figure S3. Chromatogram showing separation of (±)-mephedrone (used for preparation of calibration standards and quality control samples) into its enantiomers on LC-MS; Figure S4. Chromatogram showing separation of (±)-mephedrone (used for administration) into its enantiomers on HPLC-DAD. A superimposed chromatogram in orange shows one of the collected fractions.
