# Supplementary material for: Pharmacokinetics of Mephedrone Enantiomers in Whole Blood after a Controlled Intranasal Administration to Healthy Human Volunteers
Source: Pharmaceuticals (Basel). 2020 Dec 23;14(1):5. doi: 10.3390/ph14010005 (PMC7822411; doi:10.3390/ph14010005)
Supplement: Supplementary file 1 [file pharmaceuticals-14-00005-s001.pdf]

Pharmacokinetics of mephedrone enantiomers in whole blood after a controlled intranasal administration to healthy human volunteers

Joanna Czerwinska [a], Mark C. Parkin [a, b], Agostino Cilibrizzi [c], Claire George [d], Andrew T. Kicman [a], Paul I. Dargan [e, f], Vincenzo Abbate [a, *]

[a] King's Forensics, Department of Analytical, Environmental and Forensic Sciences, King's College London, London, UK

[b] Eurofins Forensic Services, Toxicology Department, Teddington, UK

[c] Institute of Pharmaceutical Science, King's College London, London, UK

[d] Alere Toxicology (now part of Abbott), UK

[e] Clinical Toxicology, Faculty of Life Sciences and Medicine, King's College London, London, UK

[f] Clinical Toxicology, Guy's and St Thomas' NHS Foundation Trust and King's Health Partners, London, UK

*Correspondence to: Dr Vincenzo Abbate, King's College London, Analytical, Environmental and Forensic Sciences, 150 Stamford Street, London, SE1 9NH, UK

E-mail: vincenzo.abbate@kcl.ac.uk

Keywords: mephedrone; enantiomers; whole blood; pharmacokinetics; intranasal administration

1 Validation procedures

1.1 Selectivity

Selectivity was assessed by analysing six blank matrix samples collected from drug-free female (n=3) and male (n=3) donors.

1.2 Linearity

Matrix-matched calibration curve was prepared by spiking drug-free whole blood with appropriate working solutions containing mephedrone. Each calibration standard was required to be within ± 15% of its target concentration, except at the LLOQ where ± 20% variation was allowed. The upper level of quantification (ULOQ) was defined as the highest concentration of the calibration standard. The correlation coefficient (r^2) of the curve had to be at least 0.990. A linear regression model with a weighting of 1/x was applied to all calibration curves.

1.3 Limit of detection (LOD) and limit of quantification (LLOQ)

The LOD in whole blood was defined as the lowest concentration where all three ions (two qualifiers and one quantifier) were present with a signal-to-noise ratio equal to or greater than 3. The LLOQ was defined as the lowest concentration at which analytes could be quantified with an acceptable precision and accuracy. The ULOQ was determined as the highest concentration of the calibration curve, which could be determined with an acceptable accuracy and precision without saturating the detector.

1.4 Precision and accuracy

Intra-day (n=6) and inter-day (n=3) precision and accuracy was determined by employing QC samples spiked at low, medium, and high concentrations. Intra-day precision was calculated using six replicates obtained on the same day which were expressed as a coefficient of variation (%CV). Accuracy was calculated by dividing the mean measured concentration at each QC level by the theoretical spiked concentration and was expressed as a percentage of the theoretical spiked concentration. Inter-day precision was evaluated for each QC level on three different days and expressed as %CV. According to the validation guidelines the mean value should be within 15% of the true value, except for the LLOQ where it should be within 20% of the true value.

1.5 Recovery and matrix effect

For recovery, blank whole blood samples (n=6) were spiked at QC Low and QC High level and were taken through extraction. In parallel, a set of blank whole blood samples (n=6) was extracted and spiked after the evaporation step at the QC Low and QC High level. Recovery was expressed as a percentage by comparing the absolute peak areas of the samples spiked before extraction with samples spiked after extraction.

For the IS-corrected matrix effect, a set of blank whole blood samples (n=6 from three female and three male donors) and a set of samples without matrix (n=6) were taken through extraction. All samples were reconstituted with a solution containing known amounts of the internal standard and mephedrone at QC Low and QC High levels. Matrix effect was evaluated by comparing peak area ratios in blank whole blood samples spiked after extraction with peak area ratios in samples without matrix spiked after extraction.

1.6 Carryover

Carryover was assessed by injecting matrix blanks after the highest calibration standard. According to the validation guidelines, carryover should not exceed 20% of the LLOQ.

2 Method validation

2.1 Selectivity

No interferences were observed in the extracted blank matrix.

2.2 Linearity

Mean linearity of $r^2 > 0.999$ was achieved for both enantiomers in all three validation runs. Figure S1 shows example calibration curves obtained for R-MEPH and S-MEPH.

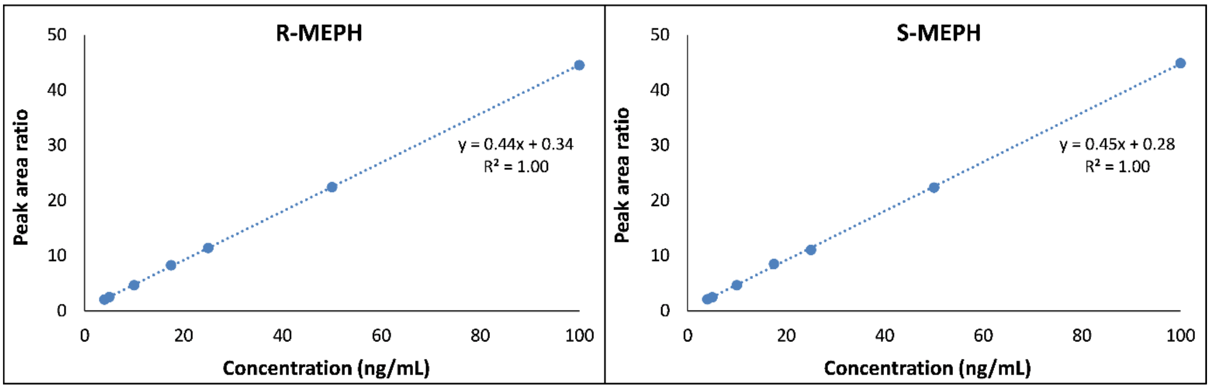

Figure S1. *Example calibration curves obtained for R-MEPH and S-MEPH*

2.3 LOD and LLOQ

LOD of 2 ng/mL and LLOQ of 8 ng/mL was achieved for both enantiomers. Table S1 shows calibration parameters for both analytes.

Table S1. *LOD, LLOQ, calibration range and calibration parameters for R-MEPH and S-MEPH in human whole blood (NaF/KOx)*

**Analyte**	**LOD** (ng/mL)	**LLOQ** (ng/mL)	**Range** (ng/mL)	**Intercept ± SD** (n=3)	**Slope ± SD** (n=3)	**r^2 ± SD** (n=3)
**R-MEPH**	2	8	8-200	9.6 ± 1.8	13.7 ± 0.9	0.999 ± 0.001
**S-MEPH**	2	8	8-200	8.9 ± 1.1	13.7 ± 0.9	0.999 ± 0.001

2.4 Precision and accuracy

Intra-day and inter-day precision and accuracy results, summarised in Table S2, were found to be within the acceptance criteria. The intra-day accuracy for both enantiomers was within ± 12% of the target concentration (101-112% for R-MEPH and 101-110% for S-MEPH). The intra-day precision was < 5.5% and ranged from 0.96-5.46% for R-MEPH and 1.13-4.70% for S-MEPH. Inter-day precision and accuracy results were acceptable over the validated range with %CV ≤ 4.35% and accuracy within ± 10% of the target concentration.

Table S2. *Precision and accuracy at QC Low, QC Med and QC High for R-MEPH and S-MEPH in human whole blood (NaF/KOx); * average value of 18 measurements over 3 days*

**Analyte**	**True value** (ng/mL)	**Mean** (ng/mL), **%CV, % accuracy**			
		**Day 1** n=6	**Day 2** n=6	**Day 3** n=6	**Inter-day** n=18 *

**R-MEPH**	10	10.7<br>0.96%<br>107%	10.5<br>3.67%<br>105%	10.1<br>2.61%<br>101%	10.4<br>3.58%<br>104%
	40	44.3<br>1.74%<br>111%	43.0<br>1.24%<br>108%	41.0<br>1.21%<br>102%	42.7<br>3.54%<br>107%
	150	168<br>5.46%<br>112%	167<br>2.48%<br>111%	161<br>3.87%<br>107%	165<br>4.35%<br>110%
**S-MEPH**	10	10.4<br>2.83%<br>104%	10.5<br>2.75%<br>105%	10.1<br>1.28%<br>101%	10.3<br>2.98%<br>103%
	40	42.8<br>3.64%<br>107%	41.9<br>1.58%<br>105%	41.0<br>1.73%<br>102%	41.9<br>3.02%<br>105%
	150	165<br>4.70%<br>110%	165<br>3.33%<br>110%	161<br>1.13%<br>107%	164<br>3.49%<br>109%

2.5 Recovery and matrix effect

As shown in Table S3, recovery for R-MEPH was 82.4 ± 4.09% and 84.5 ± 7.69% at QC Low and QC High, respectively. For S-MEPH, recovery of 71.3 ± 7.83% and 85.9 ± 7.67% at QC Low and QC High, respectively, was achieved. IS-corrected matrix effect values were within ± 2.5% at both QC levels.

Table S3. *Analyte recovery and matrix effect for R-MEPH and S-MEPH at QC Low and QC High in human whole blood (NaF/KOx)*

**Analyte**	**Recovery (%CV), n=6**		**Matrix Effect (%CV), n=6**	
	**QC Low**	**QC High**	**QC Low**	**QC High**
**R-MEPH**	82.4% (4.1%)	84.5% (7.7%)	102% (3.2%)	98.3% (1.8%)
**S-MEPH**	71.3% (7.8%)	85.9% (7.7%)	102% (7.7%)	97.5% (5.2%)

2.6 Carryover

Carryover was not observed.

3 Chemical structure of mephedrone and methcathinone

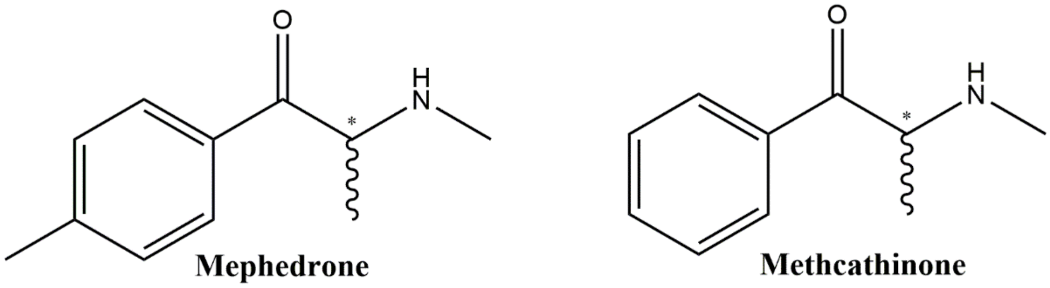

Figure S2. *Chemical structure of (±)-mephedrone and (±)-methcathinone*

4 Chiral separation of mephedrone

Figure S3 and Figure S4 show chromatographic separation of (±)-mephedrone into *R*-(+)-mephedrone and *S*-(-)-mephedrone on LC-MS and HPLC-DAD, respectively. Racemic mephedrone purchased from Sigma-Aldrich as a 1 mg/mL certified reference solution was used for the preparation of calibration standards and quality control samples (separation shown in Figure S3) whereas racemic mephedrone powder purchased from Chiron was used for the administration (separation shown in Figure S4). Retention time differs slightly between the two figures because of different void times on the analytical systems (LC-MS and HPLC-DAD). Peak area ratio of *S*-(-)-mephedrone to *R*-(+)-mephedrone is 1.02 in Figure S3 and 1.01 in Figure S4, demonstrating racemic nature of mephedrone.

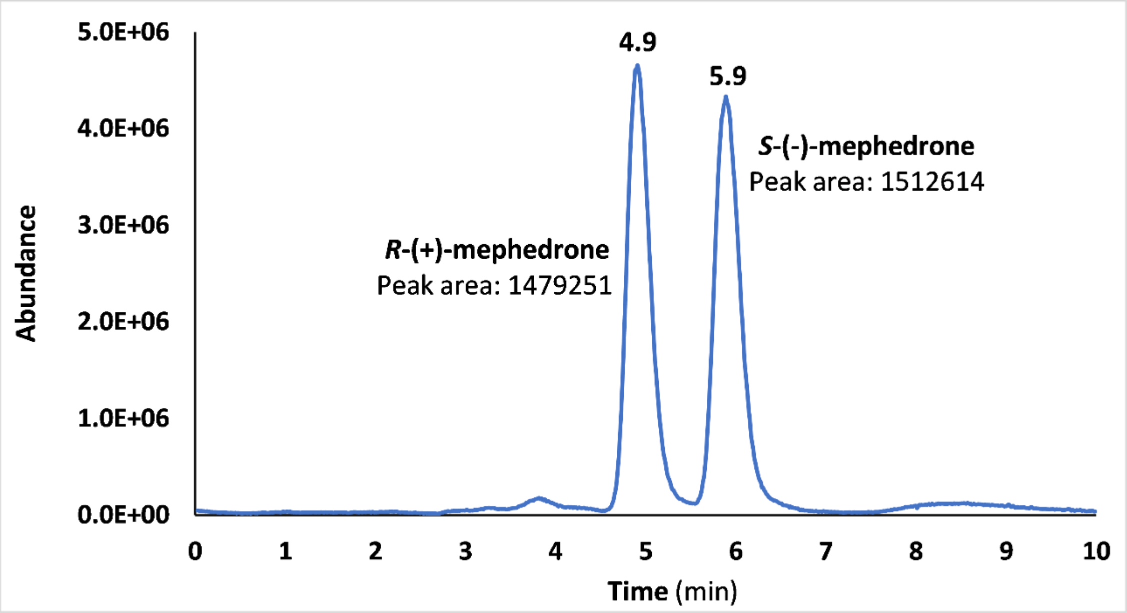

Figure S3. *Chromatogram showing separation of (±)-mephedrone (used for preparation of calibration standards and quality control samples) into its enantiomers on LC-MS*

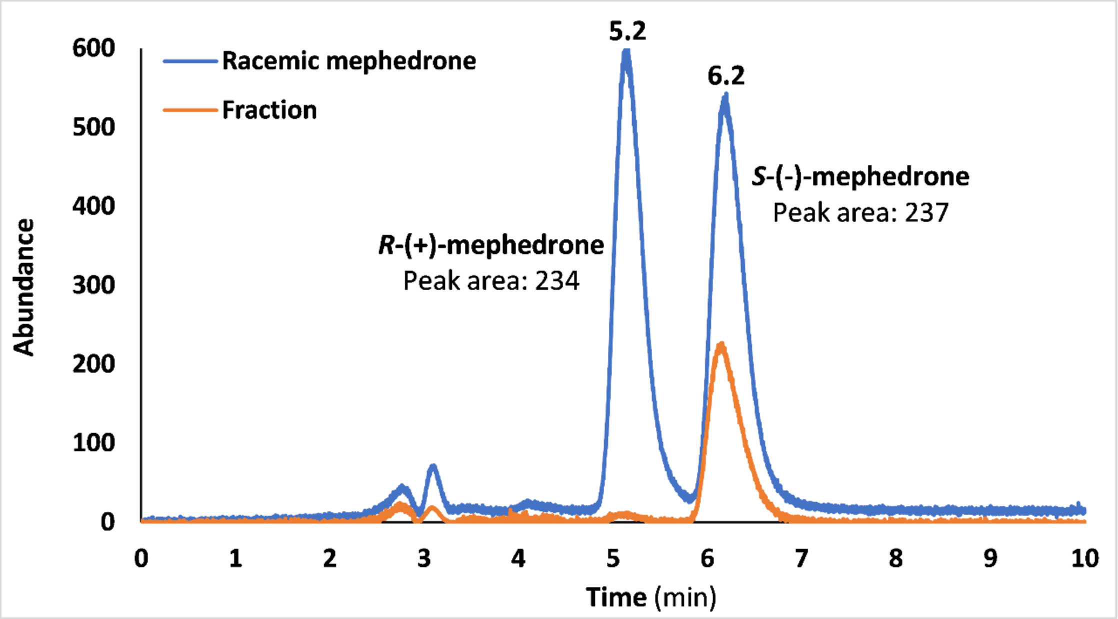

Figure S4. *Chromatogram showing separation of (±)-mephedrone (used for administration) into its enantiomers on HPLC-DAD. A superimposed chromatogram in orange shows one of the collected fractions*
